# Supplementary material for: Conversion of CO2 into Chloropropene Carbonate Catalyzed by Iron (II) Phthalocyanine Hypercrosslinked Porous Organic Polymer
Source: Molecules. 2020 Oct 9;25(20):4598. doi: 10.3390/molecules25204598 (PMC7587207; doi:10.3390/molecules25204598)
Supplement: Supplementary file 1 [file molecules-25-04598-s001.pdf]

Conversion of CO_2 into chloropropene carbonate catalyzed by iron (II) phthalocyanine hypercrosslinked porous organic polymer

Eva M Maya,[1,*] Antonio Valverde-González[1] and Marta Iglesias[1]

Figure S1. FT-IR and Raman spectra of **FePc-POP** ………..…….…….…………..2

Figure S2. UV-vis-NIR spectrum of **FePc-POP** ……………………………………….2

Figure S3. ^{13}C-NMR spectrum of **FePc-POP**……………………….….…….……..…..2

Figure S4. TGA of **FePc-POP** ………………………………………………………..3

Figure S5. N_2 adsorption/desorption isotherms of **FePc-POP** ……………..…….….3

Figure S6. SEM and EDX images of **FePc-POP** ……………..…..…………..…………4

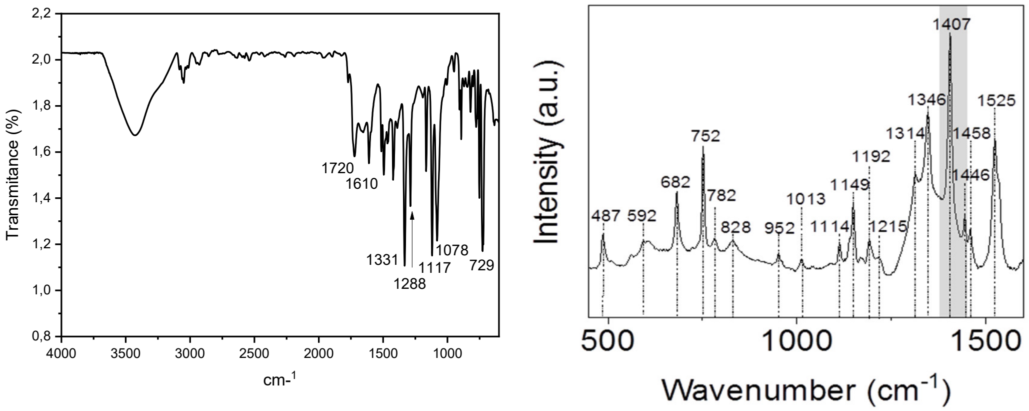

Figure S1. FT-IR spectrum (**left**) and Raman spectrum (**right**) of **FePc-POP**

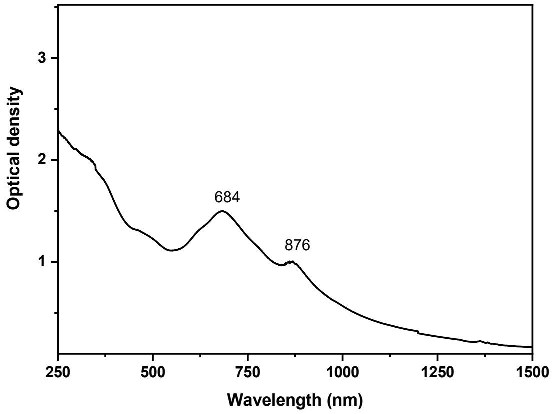

Figure S2. UV-vis-NIR spectrum of **FePc-POP**

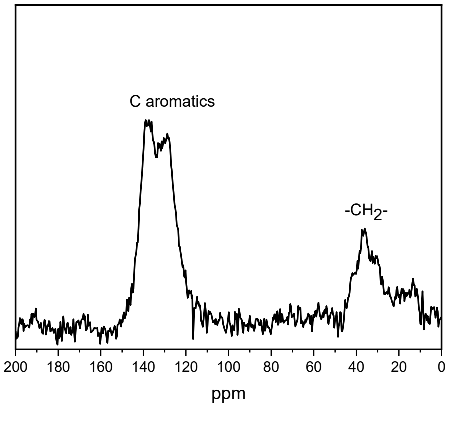

Figure S3. ^{13}C-NMR spectrum of **FePc-POP**

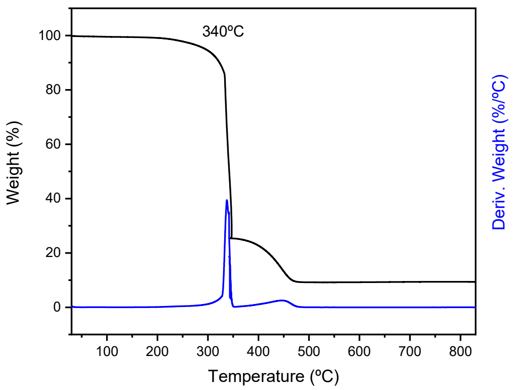

Figure S4. TGA of **FePc-POP**

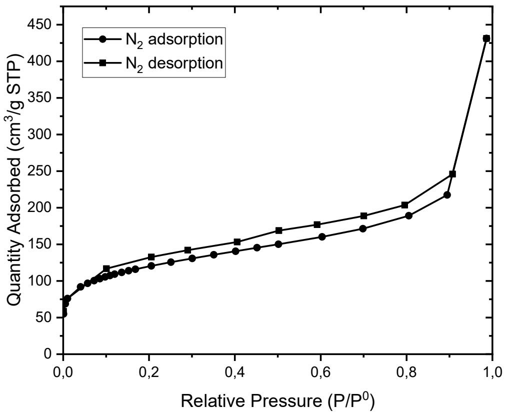

Figure S5. N_2 adsorption/desorption isotherms of **FePc-POP**

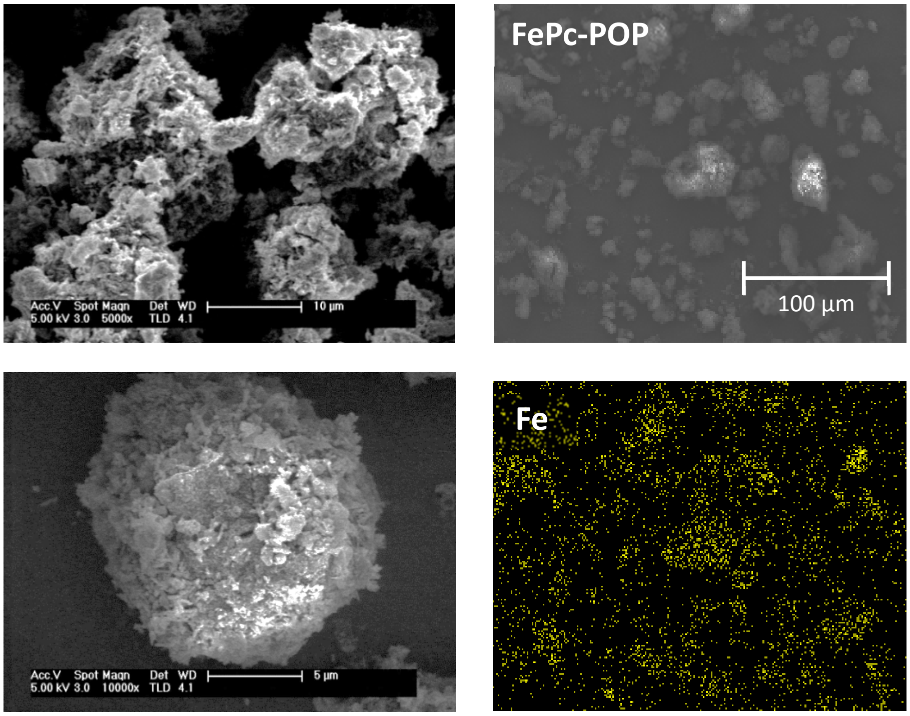

Figure S6. SEM images (**left**) and EDX analysis (**right**) of **FePc-POP**
